# Microinjection Method for Analyzing Zebrafish Early Stage Oocytes

**DOI:** 10.3389/fcell.2021.753642

**Published:** 2021-11-08

**Authors:** Manami Kobayashi, Allison Jamieson-Lucy, Mary C. Mullins

**Affiliations:** Department of Cell and Developmental Biology, University of Pennsylvania Perelman School of Medicine, Philadelphia, PA, United States

**Keywords:** oocytes, balbiani body, zebrafish, microinjection, bucky ball, oogenesis

## Abstract

Maternal factors which accumulate and establish oocyte polarity during the early stages of oogenesis play key roles in embryonic development, as well as germ cell formation. However, vertebrate oogenesis, especially early stages of oogenesis, is not well understood due to the difficulty of accessing these oocytes and the lack of analytical methods. Here, we report on a microinjection method for analyzing zebrafish early-stage oocytes and some artifacts to be aware of when performing oocyte injections or analyzing oocytes. Using this method, we successfully injected mRNAs encoding fluorescent-tagged proteins into early-stage oocytes and observed subcellular localization in the live oocytes. This method is expected to advance the functional analysis of genes involved in oogenesis.

## Introduction

Germ cells are specialized cells that differentiate into oocytes or sperm to propagate the next generation. Oocytes and sperm are generated through the processes of oogenesis and spermatogenesis, respectively, important fields in reproductive and developmental biology. Compared to spermatogenesis, vertebrate oogenesis has been little studied in part due to the inaccessibility of early stage oocytes in the ovary, as well as the lack of tools to manipulate these oocytes combined with more difficult genetic analysis. The importance of oogenesis is evident by the unusually large size of mature oocytes and eggs, which vary in diameter from 1 mm in the frog *Xenopus laevis*, 500 microns in the zebrafish, to 120 microns in human (Hirsch et al., 2002; Kimmel et al., 1995; Griffin et al., 2006). The large oocyte size reflects the large number of factors that are provided maternally and the embryonic processes that depend on these maternal factors. Moreover, in most vertebrates, cell polarity of the oocyte and egg determines the axes of the embryo, as well as germ cell formation (Heasman et al., 1984; Marlow and Mullins, 2008; Bontems et al., 2009).

Zebrafish is a good model system to study vertebrate oogenesis. Zebrafish adult females exhibit an asynchronous ovary containing all developmental stages of oocytes (Selman et al., 1993). The developmental stages of zebrafish oocytes are classified into five stages based on the size and the cellular structures of the oocyte (Selman et al., 1993). The first asymmetry found in stage I oocytes is the mitochondrial-rich structure called the Balbiani body, which is postulated to establish oocyte polarity (Dosch et al., 2004; Marlow and Mullins, 2008). This structure is conserved from insects to mammals (Jamieson-Lucy and Mullins, 2019). Another advantageous feature of the zebrafish is that juveniles have a bipotential gonad that initially develops as an ovary (Takahashi., 1977; Wang et al., 2007). Oocytes persist in fish that will become females, while oocytes are eliminated by apoptosis in gonads that transition into a testis (Uchida et al., 2002). The juvenile ovary mainly contains early stage oocytes. The body length of the juvenile fish correlates with the developmental stage of oocytes in the gonad, as described previously (Maack and Segner., 2003; Parichy et al., 2009; Elkouby and Mullins, 2017). These features allow us to obtain oocytes of particular developmental stages of interest, especially early stage oocytes.

We have developed a technique to microinject early stage oocytes, providing a useful approach to analyze the function of a gene of interest. Injecting mRNA encoding a fluorescent-tagged protein is a fast and easy way to study its subcellular localization and investigate functional domains for localization and interaction with related components. Furthermore, overexpression or knock-down experiments by injecting mRNA or anti-sense morpholinos (Nasevicius and Ekker, 2000), respectively, can provide important results on the physiological function of the gene of interest through phenotypic analysis.

**FIGURE 1:**
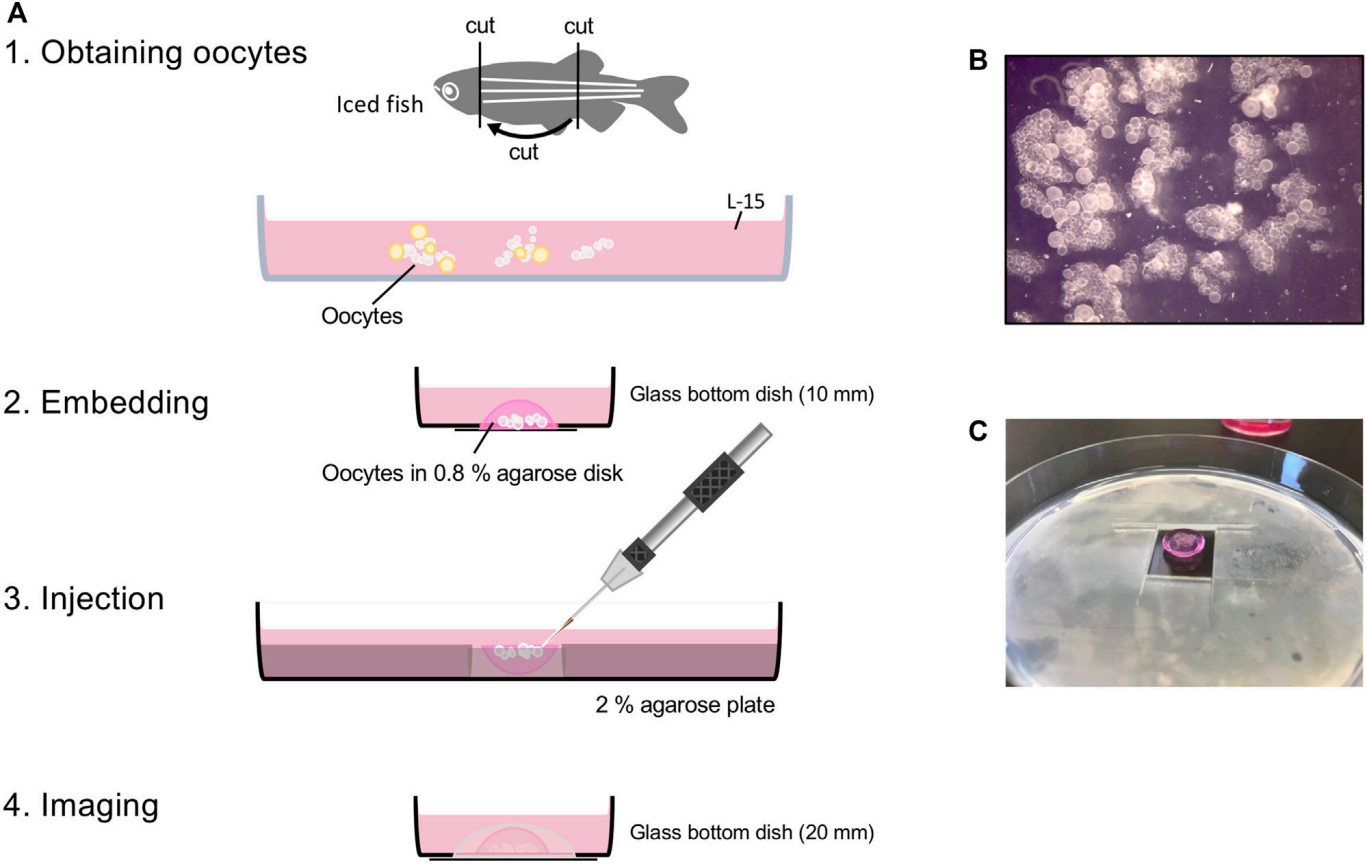
Workflow of oocyte injection. **(A)** Obtaining oocytes from juvenile or adult female fish. Embedding small clumps of ovary that has only stage I oocytes in 0.8% agarose disk **(B)**. For injection, 0.8% agarose disk is placed in the hole of the 2% agarose plate, so the oocytes face up **(C)**. After injection, flip 0.8% agarose disk so oocytes face down onto glass bottom dish for imaging.

**FIGURE 2:**
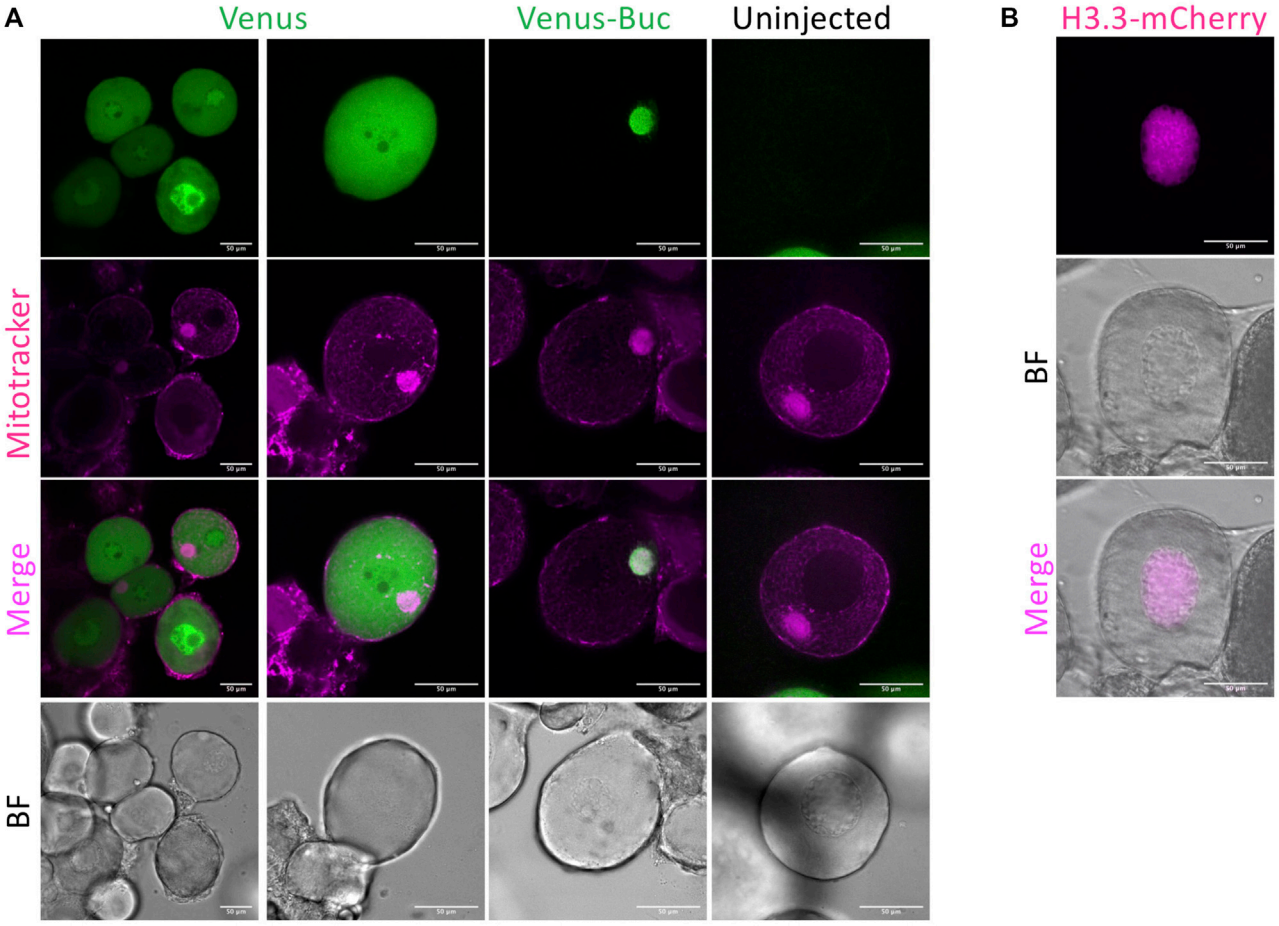
Representative data of oocyte injection. **(A)** *venus* and *venus-buc* mRNA injected oocyte. Mitotracker Red was used for staining mitochondria and the mitochondrial-rich structure called the Balbiani body (Bb) in stage 1 oocytes. Ubiquitous fluorescence was observed in *venus* mRNA injected oocytes, and Venus-Buc was detected localized to the Bb in *venus-buc* mRNA injected oocytes but not in uninjected ones. **(B)** *H3.3-mcherry* injected oocyte. Histone H3.3 is a nuclear protein localized to the nucleus. H3.3-mCherry was detected in the nucleus of an *H3.3-mcherry* injected oocyte. BF = bright field. Scale bar = 50 μm.

In zebrafish, injection of large later stage oocytes (>200 μm) was performed in several studies (Nair et al., 2013; Xie et al., 2016). However, it is much more difficult to inject early stage oocytes because they are very small (40–100 μm) and an effective injection method that achieves high injection success and survival rate had not been established. Here we provide a detailed protocol for microinjecting zebrafish early stage oocytes (Figure 1A). Using this method, we successfully injected early stage oocytes with mRNAs encoding fluorescent-tagged proteins and observed subcellular localization in the live oocytes (Figure 2).

## Materials and Equipment

• Juvenile fish (∼5–6 weeks post fertilization, standard length (SL) 10–15 mm) or Adult females
• Ice bucket
• Stereomicroscope (with high magnification 80×–100× for injection)
• Forceps (Dumont #5)
• Single edge Razor Blades (ATANLEY, #9 Steel Back Razor blades)
• Vannas spring scissors (FTS #15000-00)
• 100 mm Plastic petri dish
• 100 mm Glass petri dish
• 35 mm Glass bottom dish, 10 mm microwell (MatTek, P35G-1.5-10-C)
• 35 mm Glass bottom dish, 20 mm microwell (MatTek, P35G-0-20-C)
• Leibovitz’s L-15 medium (Gibco, #11415064, with phenol Red)
• Glutamax 100× (Gibco, #35050-061)
• Antibiotic-Antimycotic 100× (Gibco, #15240096)
• 1xPBS (pH 7.4)
• Agarose (Ronza, SeaKem LE Agarose)
• 0.8% agarose in 1xPBS, melted (kept in 42°C water bath)
• Water bath at 42°C
• 20–200 μl pipette tips
• 20–200 μl pipette tips (with the tip cut off about 1.5 cm)
• 10 μl Gel loading tips (Basix, #13611102)
• P200 pipette
• P10 pipette
• 2% agarose plate/1xPBS (number of injection conditions)
• Spatula
• Needle Puller (Sutter Instrument)
• Glass capillary needles with filament (World Precision Instruments, TW100F-4, Long taper)
• 1 M KCl
• 0.5% phenol Red/DPBS (Sigma, #P0290)
• Micromanipulator (Sutter Instrument)
• Microinjector (Sutter Instrument, XenoWorks Digital Microinjector)
• Mitotracker Red CMXRos (Molecular Probes, #M7512)
• DiOC6 (Molecular Probes, #D273)
• Foil/opaque box
• 28°C incubator
• Spinning disk confocal microscope (Olympus IX81 inverted microscope)

*Note:* Dilute Antibiotic-Antimycotic 100x (Gibco, #15240096) to 1× in Leibovitz’s L-15 medium (Gibco, #11415064, with phenol Red). L-15 medium (Gibco, #11415064, with phenol Red) contains L-glutamine, but L-glutamine is not stable. Add 100x Glutamax (Gibco, #35050-061) to 1× if needed. We also recommend adding 100× Glutamax to 1× to L-15 medium without L-glutamine each time.

## Methods

### Obtaining Oocytes

1. Euthanize fish in ice-cold water.

*Note:* To complete this experiment during the day, we recommend starting the dissection in the morning. Euthanize the fish in small batches, dissecting a few at a time to obtain fresh oocytes. Prepare at least one ovary per injection condition.

2. Place the euthanized fish on a plastic petri dish and remove the head and tail using a razor blade.
3. Pick up the trunk piece and open the body cavity along the ventral midline using forceps and a spring scissors (Figure 1A).
4. Put the trunk in a glass petri dish with 1xPBS and remove the digestive system, then pull out the swim bladder using forceps. The ovary should come out with the swim bladder (Supplementary Movie S1).
*Note:* It is easier to see the fish with an overhead (incident) light source instead of transmitted light. If you use juvenile fish and the gonad is a narrow tube of mottled milky white material, that is a testis.
5. Remove the ovary from the swim bladder and clean off the skin, connective tissue, veins, and shiny droplets of fat.
6. Place the ovary in a glass petri dish with L-15 medium.
7. Under the microscope, remove late stage oocytes (Supplementary Movie S2, adult ovary).
*Note:* Injecting works best with a small clump of the ovary that has only stage 1 oocytes (Figure 1B), and does not work well with single isolated oocytes. Any oocyte that is opaque (stage III) or looks like it has a grainy texture (early stage III) should be removed. They restrict access to the smaller stage I oocytes and when broken, their yolk makes the conditions for the oocytes around them poor. Also, the connective tissue surrounding oocytes interferes with injecting them, but it does improve oocyte survival.
8. Put a drop of L-15 media on the glass of the glass bottom 10 mm microwell dish.
9. Transfer clumps of stage 1 oocytes to the drop of L-15 media in the glass bottom dish.

### Embedding Oocytes

1. Remove as much L-15 media as possible using a P200 micropipetter.
2. Arrange oocytes in the center of the glass bottom dish using forceps and remove the rest of the L-15 media from the edge of the dish using a 10 μl gel loading tip (Supplementary Movie S3).
3. Immediately add one drop of molten 0.8% agarose from the edge of the glass bottom well using a p200 tip with the tip cut off and quickly remove the molten agarose if oocytes start to float (Supplementary Movie S4).
*Note:* After removing L-15 media, this step must be done quickly so that the oocytes do not dry up, but do carefully to keep the oocytes on the dish bottom. 0.8% agarose solidifies really fast, so a water bath is essential. Change the cut-off p200 tip, if the agarose gel solidifies in the tip.
4. Keep adding agarose drop by drop until the oocytes are secured in a dome of the agarose.
5. Cover the embedded oocytes with L-15 media while you embed oocytes for other injection conditions (Figure 1A).
*Note:* You can take breaks here. Put the embedded oocytes in the incubator (28°C) until the next step. The L-15 media with phenol red is absorbed into the agarose over time and makes it easier to identify the oocyte agarose disk. It is helpful in later steps.
6. Cut a hole (1.5 cm × 1.5 cm) in the middle of the 2% agarose plate (Figure 1C).
7. Loosen the agarose disk with embedded oocytes from the edge of the glass bottom dish using the spatula and scoop it up with the spatula.
8. Flip the agarose disk so that the oocytes face up and place it in the hole in the 2% agarose plate (Figure 1C).
*Note:* Oocytes should be embedded in the agarose securely for effective injection but gently for survival. Also, to maintain the dome shape of the agarose disk after it is flipped, a certain strength of agarose is required. Change the agarose percentage depending on the type of agarose you use. We recommend that the thickness of the 2% agarose gel plate be the same or lower than that of the agarose disk for flexible access of the injection needle.
9. Add molten 0.8% agarose around the oocyte-embedded agarose disk to secure it in place.
*Note:* Please be careful not to cover the surface of the oocyte-embedded agarose disk with 0.8% agarose at this step as it will hinder injecting the oocytes.
10. Cover the oocytes with L-15 media.
11. Put them in the incubator (28°C) until the injection set up is prepared.

## Injecting Oocytes

1. Prepare the injection mix.
  o 0.1M KCl works well.
  o 0.01% Phenol red is helpful to see that an oocyte has been injected, but it can be left out.
  o Use a high concentration of mRNA (final 200–800 ng/μl).
2. Load a needle and set up the micromanipulator; break the needle tip as small as possible.
*Note*: The size and shape of the injection needle tip is important for needle penetration and minimizing damage to the oocytes. A small tip (with the tip angled is even better) helps in injecting small oocytes. Otherwise, it is difficult for the needle to penetrate into the oocyte and the oocyte will die due to injection damage. We use thin wall, long taper needles with a filament (World Precision Instruments, TW100F-4).
3. Grab oocyte-embedded plates from the incubator. Inject the oocytes at the surface of the agarose with the smallest amount possible (Supplementary Movie S5).
*Note*: The recommended amount of injection mix is about the same as the diameter of the oocyte nucleus. Too large a volume injected will make the oocytes explode. Test the injection pressure level on stage 2 oocytes first, so as not to lose good stage 1 oocytes. If the oocytes are more than a few oocyte-diameters deep in the agarose, it will not be possible to visualize them on the confocal microscope and so it is not useful to inject them. To inject a small amount of injection mix into oocytes with small needle tip, we use a digital microinjector.
4. Return plates to the 28°C incubator after injection.
*Note*: It takes about an hour per injection condition (more than 50 oocytes can be injected per hour) – take breaks so you do not wear out your eyes, back, and fingers.
5. When all condition are finished, take plates out and free the agarose disk with embedded oocytes from the 2% agarose plate using a spatula.
6. Scoop up the agarose disk with the spatula and flip it oocytes-down onto the glass bottom dish with a 20 mm microwell.
7. Add molten 0.8% agarose around the edges to secure the agarose disk in place (Figure 1A).
8. Cover with L-15 media and incubate the oocytes (28°C), protected from light by keeping the samples in an opaque box until imaging.
*Note*: Imaging about 12 h later works well. GFP expressed from injected mRNA can be detected as soon as 3 h post injection.

## Mitochondrial Staining and Imaging

1. Incubate 30 min with Mitotracker.
*Note*: We dilute Mitotracker (1:2000) with L-15 media. Stain after injection or before imaging.
2. Rinse with L-15 media.
3. Incubate at 28°C until imaging.
4. Image with a spinning disk confocal microscope.

*Note*: The spinning disk microscope is suitable for imaging thick live samples because of its fast imaging speed, which minimizes damage to the live sample.

## Results

In this section, we show representative results obtained using this method and some artifacts to be aware of when performing oocyte injections.

### Fluorescent Dye and mRNA Injection

We injected mRNAs encoding fluorescent-tagged proteins and observed their expression in early stage oocytes. Mitotracker was used for staining mitochondria and the mitochondrial-rich structure called the Balbiani body (Bb) in stage 1 oocytes (Kosaka et al., 2007). Bucky ball (Buc) protein resides within the Bb and a transgenic Buc-GFP fusion protein also localizes to the Bb (Riemer et al., 2015). Ubiquitous fluorescence was observed in *venus* mRNA injected oocytes, and Venus-Buc was detected localized to the Bb in *venus-buc* mRNA injected oocytes but not in uninjected ones (Figure 2A). In *H3.3-mCherry* mRNA injected oocytes, a nuclear protein histone H3.3 localized to the nucleus (Figure 2B).

### Autofluorescence of the Bb From Damaged Oocytes

**FIGURE 3:**
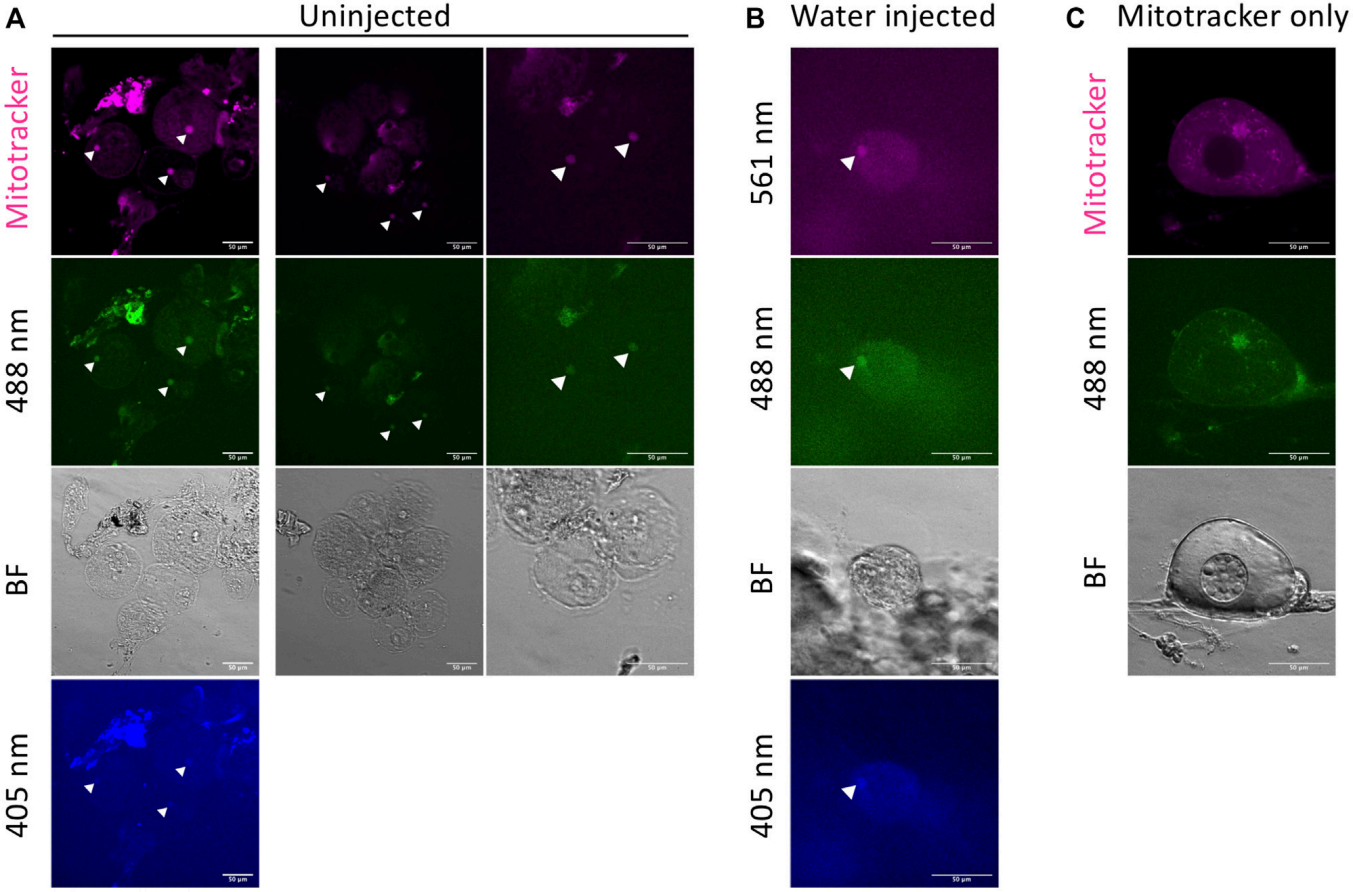
Autofluorescence in damaged oocytes. Autofluorescence of the Bb is observed in damaged oocytes with Mitotracker **(A)**, and damage-induced oocytes by water injection without Mitotracker **(B)**. Arrow heads indicate the autofluorescence from the Bb. The autofluorescence is detectable at 561, 488, and 405 nm wavelength channels. **(C)** Unexpected fluorescence in healthy oocytes. This is likely caused by remaining fluorescent dye (in this case, DiOC_6_ for 488 nm). BF = bright field. Scale bar = 50 μm.

We observed autofluorescence of the Bb (likely from mitochondria) in damaged oocytes (Figure 3). Under stress conditions, mitochondria are known to be a source of autofluorescence (Monici, 2005). We observed Bb autofluorescence in damaged oocytes (Figure 3A) and damage-induced oocytes by water injection (Figure 3B). The autofluorescence is weak but detectable at 561, 488, and 405 nm wavelength channels. This can result in false positive signals. Damaged oocytes appear ovoid, flat and dark looking (Figure 3). Select healthy oocytes that are clear and round shaped (Figure 2) for imaging. Also, if using mitotracker (or DiOC_6_) for mitochondrial staining, mitochondrial fiber structure is readily apparent in healthy oocytes.

Another artifact that we identified was persistence of DiOC6 dye in previously used glass bottom dishes. In these cases, we sometimes observed unexpected fluorescence in healthy oocytes (Figure 3C). This is likely caused by remaining fluorescent dye (in this case, DiOC_6_ for 488 nm). In this method, we can reuse the glass bottom dish because oocytes are embedded in the agarose gel disk and can be removed after imaging. However, even with well cleaned dishes, we could detect the previously used dye signal after a long incubation in the dish. So we recommend using a new dish or only using the same dyes in a given dish.

## Discussion

We successfully injected mRNA into zebrafish early-stage oocytes and observed its expression in live oocytes. Injecting stage I oocytes is challenging, since they are very small (40–100 μm), and achieving a high injection success rate and survival rate had been an obstacle to using this method. In our method, using early-stage oocytes in a small clamp with connective tissue yielded effective injection rates (more than 50 oocytes per hour) and a high survival rate (Figure 2).

This oocyte injection technique is a fast and easy way to study a gene of interest, in contrast to the more time-consuming methods of generating transgenics or using CRISPR/Cas9 knock-in technology. Producing mRNA *in vitro* from a plasmid construct to inject is a quick and good way to investigate a protein’s subcellular localization, as well as, for example, defining the functional subdomains for localization and interaction with other components. Moreover, the injection technique can be used in combination with existing mutant and transgenic lines, allowing a variety of experimental designs.

Developing a long-term culture technique after injection would provide even more advantages to study oogenesis. A culture method in which zebrafish primary oocytes develop to stage II still requires further development. While we have not performed time-lapse imaging of the injected oocytes, it would provide real-time dynamics of protein localization. Combined with super-resolution live imaging techniques, one could observe with high precision the dynamic behavior of a protein of interest during oogenesis. Furthermore, overexpression or knock-down experiments by injecting mRNA or anti-sense morpholinos, respectively, can provide important results on the physiological function of the gene of interest through phenotypic analysis. We expect this method will advance the functional analysis of genes acting in oogenesis.

## Data Availability

The original contributions presented in the study are included in the article/Supplementary Material, further inquiries can be directed to the corresponding authors.
